# Gabapentin-Induced Mood Disorder and Psychosis in a Patient with Mild Traumatic Brain Injury: A Case Report

**DOI:** 10.51894/001c.165992

**Published:** 2026-07-31

**Authors:** Tirth Patel, Dunyue Lu

**Affiliations:** 1 College of Osteopathic Medicine Michigan State University

## 111

### Introduction

Gabapentin is widely prescribed for many clinical conditions including seizure, anxiety, neuropathic pain and is generally well-tolerated. However, psychiatric adverse effects including emotional disturbances and/or psychosis have been reported with gabapentin use. These effects may be potentiated in patients with underlying neurological vulnerability such as mild traumatic brain injury (TBI). We present a case of gabapentin-induced mood disorder and psychosis with commanding hallucinations and homicidal ideation in a patient with neuropathic pain, post-concussive syndrome and post traumatic stress disorder (PTSD) following a motor vehicle accident.

### Case Description

A 57-year-old male with no significant psychiatric history presented to the emergency department after being struck by a vehicle traveling approximately 20–25 mph. He sustained a closed head injury, left pulmonary contusion, trace pneumothorax, and seizure-like activity. Initial CT brain and cervical spine imaging were negative for acute intracranial pathology. Shortly after discharge, he developed neuropathic left lower extremity pain, post-concussive symptoms, and acute stress disorder. At two-week follow-up, gabapentin 200 mg BID was initiated for neuropathic pain. Two weeks later, the dose was increased to 300 mg BID. Shortly after, the patient developed progressive visual and auditory hallucinations, which included commanding hallucinations, delusions, dissociation, and identity disturbances. He also reported homicidal ideation, hostility and aggression. One month later, he was emergently referred to psychiatric evaluation by his primary care physician. He reported spontaneous resolution of symptoms following discontinuation of gabapentin for two occasions. Laboratory workup including metabolic panel and CT brain were unremarkable. Gabapentin was discontinued after psychiatric consultation. The patient’s mood symptoms and psychoses subsided, suggesting that mood disorder and psychosis are induced by gabapentin.

### Discussion

This case highlights that low dose of gabapentin is a potential precipitant of acute mood disturbances and psychosis, particularly in patients with underlying neuropathic pain, post-concussive syndrome and PTSD. The temporal correlation between gabapentin initiation at 200 mg BID, dose increase to 300 mg BID, symptom onset, and symptom resolution following discontinuation supports a substance-induced etiology. Clinicians should maintain heightened caution for psychiatric adverse effects when prescribing gabapentin for neuropathic pain to patients with mild TBI or trauma-related disorders and should consider alternative analgesics.

